# *Lycium barbarum* polysaccharide ameliorates the accumulation of lipid droplets in adipose tissue via an ATF6/SIRT1-dependent mechanism: *Lycium barbarum* polysaccharide ameliorate obesity

**DOI:** 10.3724/abbs.2024046

**Published:** 2024-04-11

**Authors:** Rui Zhou, Yajing Liu, Weiqian Hu, Jing Yang, Bing Lin, Zhentian Zhang, Mingyan Chen, Jingwen Yi, Cuifeng Zhu

**Affiliations:** Department of Clinical Nutrition Shenzhen Hospital of Southern Medical University Shenzhen 518000 China

**Keywords:** *Lycium barbarum* polysaccharide, lipid droplets, obesity, SIRT1, ATF6

## Abstract

Lipid droplets (LDs) are dynamic organelles that store neutral lipids and are closely linked to obesity. Previous studies have suggested that *Lycium barbarum* polysaccharide (LBP) supplements can ameliorate obesity, but the underlying mechanisms remain unclear. In this study, we hypothesize that LBP alleviates LD accumulation in adipose tissue (AT) by inhibiting fat-specific protein 27 (Fsp27) through an activating transcription factor-6 (ATF6)/small-molecule sirtuin 1 (SIRT1)-dependent mechanism. LD accumulation in AT is induced in high-fat diet (HFD)-fed mice, and differentiation of 3T3-L1 preadipocytes (PAs) is induced. The ability of LBP to alleviate LD accumulation and the possible underlying mechanism are then investigated both *in vivo* and *in vitro*. The influences of LBP on the expressions of LD-associated genes ( *ATF6* and *Fsp27*) are also detected. The results show that HFD and PA differentiation markedly increase LD accumulation in ATs and adipocytes, respectively, and these effects are markedly suppressed by LBP supplementation. Furthermore, LBP significantly activates SIRT1 and decreases ATF6 and Fsp27 expressions. Interestingly, the inhibitory effects of LBP are either abolished or exacerbated when *ATF6* is overexpressed or silenced, respectively. Furthermore, SIRT1 level is transcriptionally regulated by LBP through opposite actions mediated by ATF6. Collectively, our findings suggest that LBP supplementation alleviates obesity by ameliorating LD accumulation, which might be partially mediated by an ATF6/SIRT1-dependent mechanism.

## Introduction

Obesity is a serious global epidemic associated with increased morbidity and mortality and places a considerable burden on both individuals and public health [ 1, 2] . The WHO global report on obesity revealed overweight (BMI ≥ 25 kg/m ^2^) in 39% of males and 39% of females aged over 18 years in 2016 and obesity (BMI ≥30 kg/m ^2^) in 11% of males and 15% of females [3]. There are nearly 2 billion overweight adults globally, of whom more than 500 million are obese [World Obesity Atlas 2022. https://www.worldobesity.org/resources/resource-library/world-obesity-atlas-2022 (Accessed August 15, 2022)]. Due to the poor effects and potential adverse effects of anti-obesity drugs, there has been a growing focus on finding alternative fat-lowering nutritional therapy strategies, especially through natural dietary products [4]. Currently, most of these natural products or related food items are considered good alternatives to anti-obesity drugs [5]. Among them, *Lycium barbarum* polysaccharide (LBP), also known as “Goji berries”, is a medical plant with a long history in traditional Chinese medicine [6]. Previous studies on humans and rodents have reported that LBP has many beneficial effects, *e*. *g*., cardiovascular preservation, anticancer effects and antioxidant effects. LBP has also been reported to improve glycemic control in animals and prevent obesity [6]. Intriguingly, LBP can also reduce body fat by inhibiting the fat accumulation process [7]. However, the mechanism involved remains uncharacterized.

Lipid droplets (LDs) are dynamic subcellular organelles whose growth is strongly associated with obesity. LDs are widely distributed in different tissues, such as fat and liver, and comprise a neutral lipid core that is composed of cholesterol esters (CE) and triglycerides (TG) [8]. LDs are believed to be derived from the endoplasmic reticulum (ER), where TG is synthesized [9]. As a member of the CIDE family of proteins, fat-specific protein 27 (Fsp27) is critical for unilocular LD generation in adipocytes by regulating atypical LD fusion through directional neutral lipid transfer at the LD-LD contact site (LDCS) [10]. Fsp27 expression is upregulated in adipose tissue (AT), and Fsp27 deficiency causes a drastic reduction in lipid storage [ 11– 13] . In contrast, ectopic Fsp27 expression leads to an increase in the size of LDs [ 14– 16] . However, little is known about the role of Fsp27 in LD accumulation in AT in the context of LBP treatment.

It has long been known that excessive lipid intake induces ER stress and unfolded protein response (UPR) [17]. A previous study revealed that LBP might prevent obesity via the ERS signaling pathway [6]. Activating transcription factor-6 (ATF6), one of three ER stress transducers and a basic region-leucine zipper (bZip) transcription factor, has certain effects on lipid accumulation [18]. Under normal conditions, ATF6 remains inactive. Under ER stress, the UPR cascade that stimulates ATF6 is activated [19]. From these studies, it is likely that ATF6 may be involved in LD accumulation. Our research group reported that Equol, another natural dietary product, can attenuate atherosclerosis by inhibiting ER stress in vascular endothelial cells, which is partially mediated through downregulating ATF6 expression [20]. However, few studies have assessed the effect of LBP on ATF6 expression in AT. Furthermore, LBP is known to be a small-molecule sirtuin 1 (SIRT1) activator [ 21, 22] , requiring nicotinamide adenine dinucleotide (NAD ^+^) for its deacetylation activity [23]. The NAD ^+^-dependence of SIRT1 strongly associates its activity with cellular energy. The production of SIRT1, which is induced by caloric restriction and exercise [24], is essential for regulating lipid and glucose homeostasis [25]. Given the close connection between SIRT1 and cellular energy and energy homeostasis, SIRT1 has become a molecular target of interest for metabolic disorders, such as obesity. SIRT1 is a known negative regulator of ER stress response. Furthermore, SIRT1 has been shown to deacetylate X-box-binding protein 1 (XBP1) and suppress its transcriptional activity, thus enhancing ER stress-induced apoptosis; however, in mammals, SIRT1 also weakens PERK-eIF2α-dependent translational inhibition [ 26– 28] . However, the underlying mechanism involved in the interaction between ATF6 and SIRT1 in LBP-induced inhibition of LD accumulation has never been explored.

In this study, we found that LBP could ameliorate LD accumulation in mouse ATs and adipocytes *in vitro* through an ATF6/SIRT1-dependent mechanism.

## Materials and Methods

### Reagents

By high-performance liquid chromatography (HPLC), the purity of LBP (SP9311; Solarbio Science & Technology, Beijing, China) was determined to be >90%. Fetal bovine serum (FBS) and Dulbecco’s modified Eagle’s medium (DMEM) were supplied by Hyclone (Carlsbad, USA). Isobutylmeth-ylxanthine (IBMX), dexamethasone (DEX) and insulin were obtained from Sigma-Aldrich (St Louis, USA). The antibodies used included anti-SIRT1 (2028; Cell Signaling Technology, Danvers, USA), anti-ATF6 (PA5-20215; Thermo Fisher Scientific, Rockford, USA) and anti-Fsp27 (PA1-46128, Thermo Fisher Scientific), and anti-actin (TA-09; Zhongshan Jinqiao Biotechnology, Beijing, China). Trizol reagent was obtained from Hangzhou Bioer Technology (Hangzhou, China). *ATF6* siRNA (sense, 5′-CAGCACGUUCCUGAGGAGUUGGAUU-3′; antisense, 5′-CAGCACGUUCCUGAGGAGUUGGAUU-3′) and its control siRNA (sense, 5′-CAGUGCCCUAGUAGGUUGGGCAAUU-3′; antisense, 5′-AAUUGCCCAACCUACUAGGGCACUG-3′) were constructed by GeneCopoeia (Guangzhou, China). The pAAV-ZsGreen-shRNA-mSIRT1 ( *shSIRT1*) plasmid, p3×FLAG-ATF6 plasmid and their corresponding control vectors were purchased from Life Technology (Shanghai, China).

### Animal experiment

Eight-week-old ( *n*=20) and 4‒6-week-old ( *n*=25) male C57BL/6 mice were obtained from the Medical Experimental Animal Center of Shenzhen Hospital, Southern Medical University. The use and care of these animals strictly followed the Institutional Guidelines for the Use of Laboratory Animals, and all experiments were carried out after obtaining the approval from the Ethics Committee of Shenzhen Hospital, Southern Medical University (No. 2021-0063). All mice were housed in ventilated cages in a specific pathogen-free (SPF) animal facility with controlled environmental settings (21‒22°C, 30%‒60% humidity, 12/12-h light/dark cycle, and food and water available *ad libitum)*. Alterations in mouse activity, physical condition, and body weight (BW) were monitored.

Protocol 1: BW-matched C57BL/6 mice were randomized into 4 groups and fed with a chow diet (CHOW), a high-fat diet (HFD), a HFD supplemented at 50 mg/kg/day LBP, or a HFD supplemented with 100 mg/kg/day LBP. The CHOW diet and HFD were obtained from Medicience Ltd. (Yangzhou, China). LBP was diluted with distilled water and supplemented by gavage once a day at 8:00 am for 12 weeks. The CHOW and HFD groups were given equal volumes of distilled water. The BW was examined every week.

Protocol 2: Initially, C57BL/6 mice were fed with CHOW diet or a HFD (containing 60% fat) for two weeks. After 30 days, HFD-fed mice were gavaged with or without LBP (400 mg/kg/day) or with or without a Tail vein injection of the pAAV-ZsGreen-shRNA-mSIRT1 plasmid once every 5 days. *shSIRT1* (12.5 μg)+non-liposomal nanoparticle transfection reagent (25 μL; Entranster- *in vivo*; Engreen Biosystem, Beijing, China) mixed with 10% glucose solution was used for each injection following the supplier’s protocol [29]. The BW was measured every two days.

Mice were subjected to starvation overnight and then sacrificed. Serum samples obtained by solidification and centrifugation (3000 *g* for 10 min at 4°C) were refrigerated at –80°C until biochemical parameters were tested. After the white AT of the abdomen was carefully removed, it was weighed, photographed, and subsequently lysed using RIPA lysis buffer (20 mg tissue/300 μL RIPA) for western blot analysis. Other tissues were immediately stored in liquid nitrogen at –80°C for other assays, such as plasma TG detection and RT-qPCR. The plasma LBP concentration was determined by HPLC.

### Histological analysis

The harvested tissues were fixed with 4% paraformaldehyde and paraffin embedded. The sections (5 μm) were then stained with hematoxylin and eosin (H&E). All sections were microscopically analyzed. The size of adipocytes, indicated by the cross-sectional surface area on randomly selected H&E-stained sections, was measured using ImageJ software (version 6.0; National Institutes of Health, Bethesda, USA).

### MRI in mice

A radiologist blinded to group allocation performed MRI measurements on mice utilizing a 7T MRI scanner (70/16 PharmaScan; Bruker, Billerica, USA). Anesthesia was induced with 3% isoflurane. During the MRI scans, the mice were placed on a plastic cradle with tooth bars and plastic screws in their ear canals. Then, continuous physiological monitoring was performed using an MRI-compatible system (SA Instruments, Naples, USA). The experimental parameters were as follows: spin-echo sequence, TR/TE=150/5.8 ms; field of view (FOV)=31.50×20.25 mm ^2^; and matrix size=210×135. The AT of the entire abdomen of each mouse was covered with axial slices with a thickness of 1 mm but no spacing.

### Cell culture and intervention

3T3-Ll preadipocytes (PAs) were obtained from the American Type Culture Collection (Manassas, USA) and cultivated in DMEM supplemented with 25 mM glucose (DMEM-H), penicillin (100 U/mL), sodium pyruvate (1 mM), streptomycin (100 μg/mL), and 10% FBS in a humidified atmosphere with 5% CO _2_ at 37 °C. Two days after confluence, the cells were stimulated to induce differentiation using DMEM supplemented with 10% FBS, insulin (167 nM), DEX (1 M), and IBMX (0.5 mM) for 48 h. Then, the cells were cultured in 10% FBS/DMEM+insulin (167 nM) for an additional 48 h and then in 10% FBS/DMEM for another 96 h, after which 90% of the cells grew to mature adipocytes with accumulated LDs. All stimulations were conducted only after a period of culture of 3T3-L1 adipocytes in DMEM with no additions for the time period described [30]. 3T3-Ll PAs, differentiating adipocytes and mature adipocytes were treated with a series of concentrations (0.1, 1, 10, 25, 50, and 100 μM) of LBP for 24 h for further experiments. Rosiglitazone (ROSI), which can increase lipid accumulation and promote the differentiation of fat cells, was used as a positive control [31].

### Cell viability assay

3T3-Ll PA viability was measured using a Cell counting kit-8 (CCK-8) (MedChemExpress, Monmouth Junction, USA). The cells were seeded into a 96-well microplate (3650; Corning Life Sciences, Acton, USA) at 5×10 ^3^ cells/well and cultured for 24 h and then treated as described above. The cells were incubated at 37°C for 1 h after the addition of CCK-8 solution at 10 μL/well, after which viability was determined via absorbance measurements (450 nm) utilizing a monochromator microplate reader (Safire II; Tecan Group Ltd., Männedorf, Switzerland). The percentage of viable cells relative to the control group (set as 100%) was calculated.

### RNA interference and lentivirus transfection

The p3×FLAG-ATF6 plasmid and its corresponding control pCMV-3FLAG-7.1 vector were transfected into 3T3-Ll adipocytes via Lipofectamine 2000 (11668-027; Invitrogen, Carlsbad, USA) according to the manufacturer’s instructions. For the siRNA assay, 3T3-Ll adipocytes were transfected with the indicated siRNA via Lipofectamine 2000 for 24 h. After that, the cells were exposed to different concentrations of LBP for 24 h.

### Lipid content measurement

The intracellular TG content was assayed using a TG assay kit (GPO-POD method; Plygen Technologies Inc., Beijing, China) following the manufacturer’s instructions. The absorbance was detected at 510 nm using a fully automatic biochemical analyzer.

### Fluorescence microscopic imaging

Following the indicated treatment, 3T3-L1 adipocytes were fixed with 4% paraformaldehyde (30 min), immersed in 0.1% Triton X-100 (20 min), and blocked with 10% goat serum (1 h) at room temperature. The cells were incubated with a primary antibody against Fsp27 overnight at 4°C. The Alexa Fluor 488-conjugated anti-rabbit IgG antibody (A11008; Molecular Probes, San Jose, USA) was utilized as a secondary antibody. Lipid staining was performed using Bodipy558/568 (D3835; Molecular Probes). After nuclear counterstaining with 4′,6′-diamidino-2-phenylindole (DAPI) for 5 min at room temperature, the slides were observed using a Zeiss 200 M inverted microscope, and images were acquired with confocal laser scanning microscope (model TCS SP2; Leica Microsystems GmbH, Wetzlar, Germany).

### Western blot analysis

Mature adipocytes (1×10 ^6^) cultured in culture dishes with a size of 100 mm ^2^ were treated as indicated. Whole-cell lysis products, prepared with ice-cold RIPA buffer, were subjected to sodium dodecyl sulfate-polyacrylamide gel electrophoresis, as previously reported [32]. The samples were transferred onto a polyvinylidene fluoride (PVDF) membrane for incubation at 4°C with the appropriate primary antibody overnight. This was followed by incubation with a goat anti-mouse/rabbit IgG horseradish peroxidase-conjugated secondary antibody. A Fusion FX5 Spectra imaging system (Vilber Lourmat Inc., Collégien, France) was utilized for the detection of protein bands.

### RNA isolation and RT-qPCR

After total RNA was isolated from mature adipocytes using Trizol reagent, a 1 μg aliquot was reverse transcribed. Using an iQ5 machine (Bio-Rad, Hercules, USA), 25 μL of reaction mixture was added, and targeted genes were amplified as follows: denaturation (95°C, 30 s); 40 cycles of 95°C for 5 s and 60°C for 34 s; and extension (95°C for 15 s and 30°C for 6 s). The amplification reactions were analyzed by the comparative threshold cycle (Ct) method and were normalized against *GAPDH*, a reference control. The sequences of primers used for amplification are listed in Table 1.

**Table:** **Table 1** Sequences of primers used for RT-qPCR

Gene	Forward primer (5′→3′)	Reverse primer (5′→3′)
*SIRT1*	TAGCCTTGTCAGATAAGGAAGGA	ACAGCTTCACAGTCAACTTTGT
*ATF6*	GACAGTACCAACGCTTATGCC	CTGGCCTTTAGTGGGTGCAG
*Fsp27*	TCGTGTTAGCACCGCAGAT	GCTCTCTTCTTGCGCTGTT
*ATF6* ChIP	CACACGTTTGAAGCCAAGCT	CCCATCACTTGTAGGGTGGT
*GAPDH*	CAACTTTGGTATCGTGGAAGGAC	ACAGTCTTCTGGGTGGCAGTG

### Dual-luciferase reporter assay

Luciferase activity was measured after 48 h of LBP treatment according to the instructions of the Dual-Luciferase Reporter Assay System (Promega, Madison, USA). The potential modulation of ATF6 in the mouse *SIRT1* promoter region by LBP treatment was predicted using Genomatix, UCSC and JASPAR, three online algorithms. Based on the predicted overlapping findings, a 1.2-kb mouse *SIRT1* promoter sequence (‒2000 to +100 bp) was synthesized for cloning into the *Xho*I and *Hin*dIII sites of the pGL3-Basic vector (Life Technology), which was called the pAAV-ZsGreen-shRNA-mSIRT1 ( *shSIRT1*) plasmid. The construct obtained was further verified via DNA sequencing. Following the recommendations of Lipofectamine 2000, 3T3-L1 adipocytes were cotransfected with *shSIRT1* or pGL3-Basic-SIRT1 plasmids along with a pRL-TK plasmid (Promega), followed by the transfection of ATF6 with a siRNA or a p3×FLAG-ATF6 plasmid, respectively. The luciferase activity was measured and calculated as the ratio of firefly luciferase activity to Renilla luciferase activity. Each measurement, as well as each assay that was performed on adipocytes, was performed in triplicate.

### Chromatin immunoprecipitation (ChIP)

ChIP assay was performed with a ChIP kit (Millipore, Billerica, USA) as described in a previous study [33]. Briefly, following PA (0.2 mM) or vehicle control (0.1% ethanol) treatment for 24 h, 3T3-L1 adipocytes were subjected to fixation with 1% formaldehyde at 37°C for 10 min to cross-link the nuclear proteins to DNA. The cells were then collected for lysis with SDS lysis buffer. DNA in chromatin was sonically cleaved to 200‒1000 bp in length, followed by immunoprecipitation with 2 μg of an antibody against ATF6 (negative control: IgG). After DNA precipitation, the DNA was processed for PCR or qPCR amplification using primer pairs overlapping the *SIRT1* promoter region *ATF6* (‒1340 to ‒1333), with total DNA extracted (input) serving as a positive PCR control.

### Statistical analysis

Unless otherwise stated, data from various groups are expressed as the mean±SD. Multigroup (≥3) and intergroup comparisons were achieved by one-way ANOVA and two-tailed unpaired *t* test, respectively. The significance of the *in vivo* findings was determined by repeated-measures ANOVA. *P*<0.05 was considered statistically significant.

## Results

### LBP protects AT from lipid accumulation in a HFD-fed mouse model

**Figure 1:**
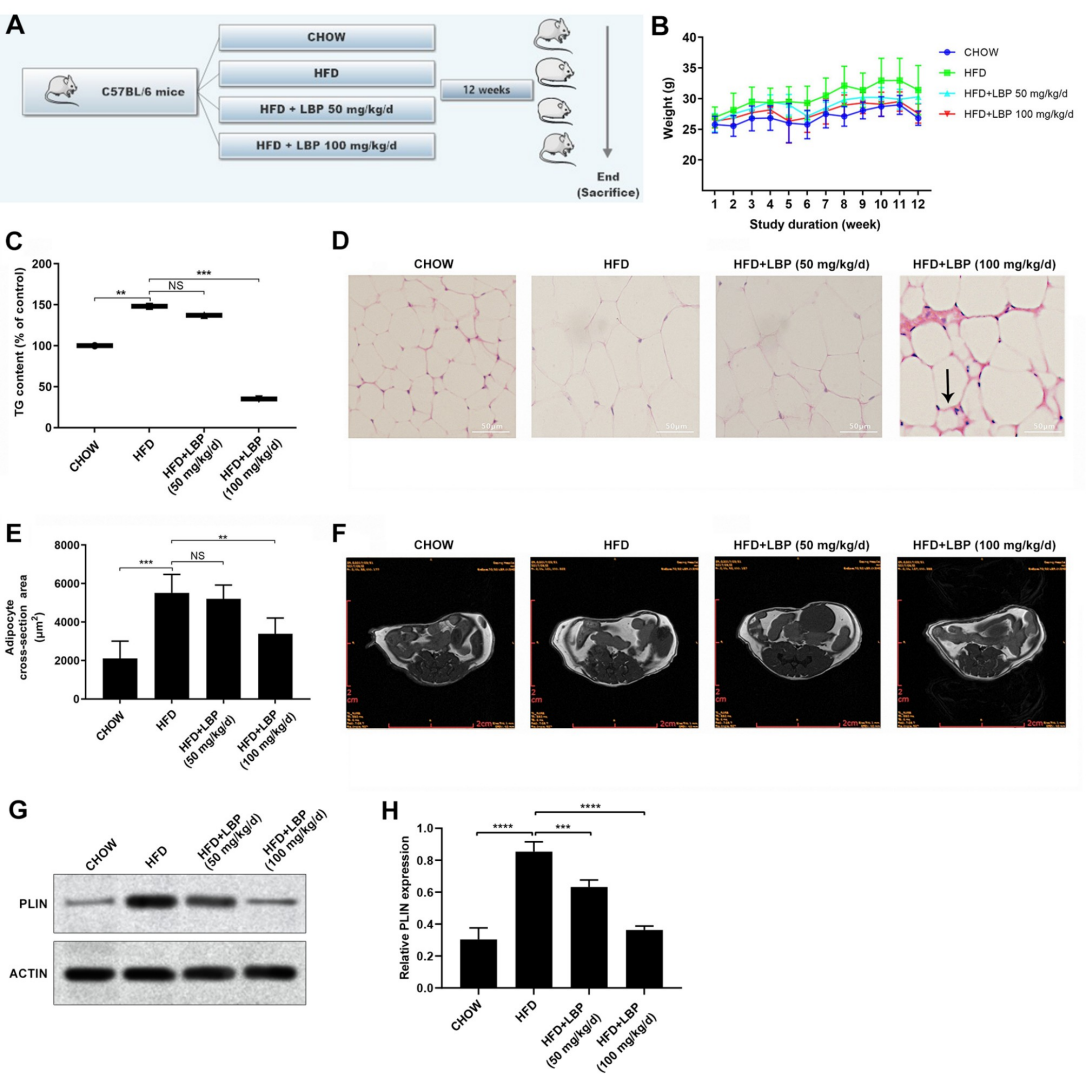
Figure 1 LBP protects adipose tissue from lipid accumulation in a HFD-fed mouse model (A) Four- to six-week-old male C57BL/6 mice were randomized into 4 groups ( n=5 for each) and fed with a chow diet (CHOW), a high-fat diet (HFD), a HFD +50 mg/kg/day LBP or a HFD+100 mg/kg/day LBP. (B) Body weight (BW) of the mice. (C) Plasma triglyceride (TG) detection with a TG content detection kit. (D) H&E staining of mouse adipose tissue specimens. Scale bar: 50 μm. (E) The size of adipocytes, indicated by the cross-sectional surface area on randomly selected H&E-stained sections, was measured using ImageJ software. (F) MR images of the different groups. (G,H) Western blot analysis of PLIN expression in mouse adipose tissue from different groups. The quantitative data obtained after three repeated measurements are presented as the mean±SD. ** P<0.01, *** P<0.001, and **** P<0.0001. No statistical significance is denoted by NS.

In this study, animals were randomized into 4 groups and fed with a CHOW diet or a HFD in the presence or absence of LBP for 12 weeks ( Figure 1A). The HFD-fed mice gained much more BW than the chow-fed mice beginning in the 8 ^th^ week, and the BW of the HFD-fed mice remained the highest among all the groups throughout the entire experimental period ( Figure 1B). At the end of the 12 ^th^ week, the BWs of the mice fed with 50 and 100 mg/kg/day LBP were 30.3±1.64 g and 27.58± 1.57 g, respectively, which were much lower than those of the HFD-fed mice (31.4± 2.4 g) ( *P*<0.05). The daily caloric intake differed insignificantly among the groups fed with a HFD in the presence or absence of LBP supplementation. Furthermore, a higher concentration (100 mg/kg/day) of LBP led to a more obvious reduction in plasma TG ( Figure 1C). As measured by H&E staining, as expected, the size of adipocytes from visceral adipose tissue (VAT) was much greater in HFD-fed mice than in CHOW diet-fed mice ( Figure 1D,E). However, the adipocytes from the LBP-treated mice had multilocular LDs that were smaller than those from the CHOW group which had larger and more unilocular LDs. Interestingly, administration of LBP also resulted in crown-like structures (CLSs), which are characteristic of infiltrating macrophages surrounded by dead adipocytes, in the AT of obese animals and humans [34]. In this study, CLSs were notably more common in the AT of mice fed with 100 mg/kg/day LBP than in that of mice fed with a HFD, whereas CLSs were not significantly different in mice fed with 50 mg/kg/day LBP ( Figure 1D). Abdominal MR images of live mice at the 11 ^th^ week further confirmed these findings ( Figure 1F). To further demonstrate the influence of LBP on lipid accumulation, we tested the expression of PLIN (a lipid droplet-associated protein) in mouse AT. PLIN expression was greater in HFD-fed mice than in CHOW diet-fed mice ( Figure 1G,H). However, administration with 50 or 100 mg/kg/day LBP attenuated the expression of PLIN in the AT of HFD-fed mice. Taken together, these findings indicate that LBP administration in HFD-fed mice alleviates lipid accumulation in AT, resulting in a decreased size and decreased number and mass of LDs in mouse AT.

### LBP inhibits lipid accumulation in AT through SIRT1 in a short-term HFD-fed mouse model

**Figure 2:**
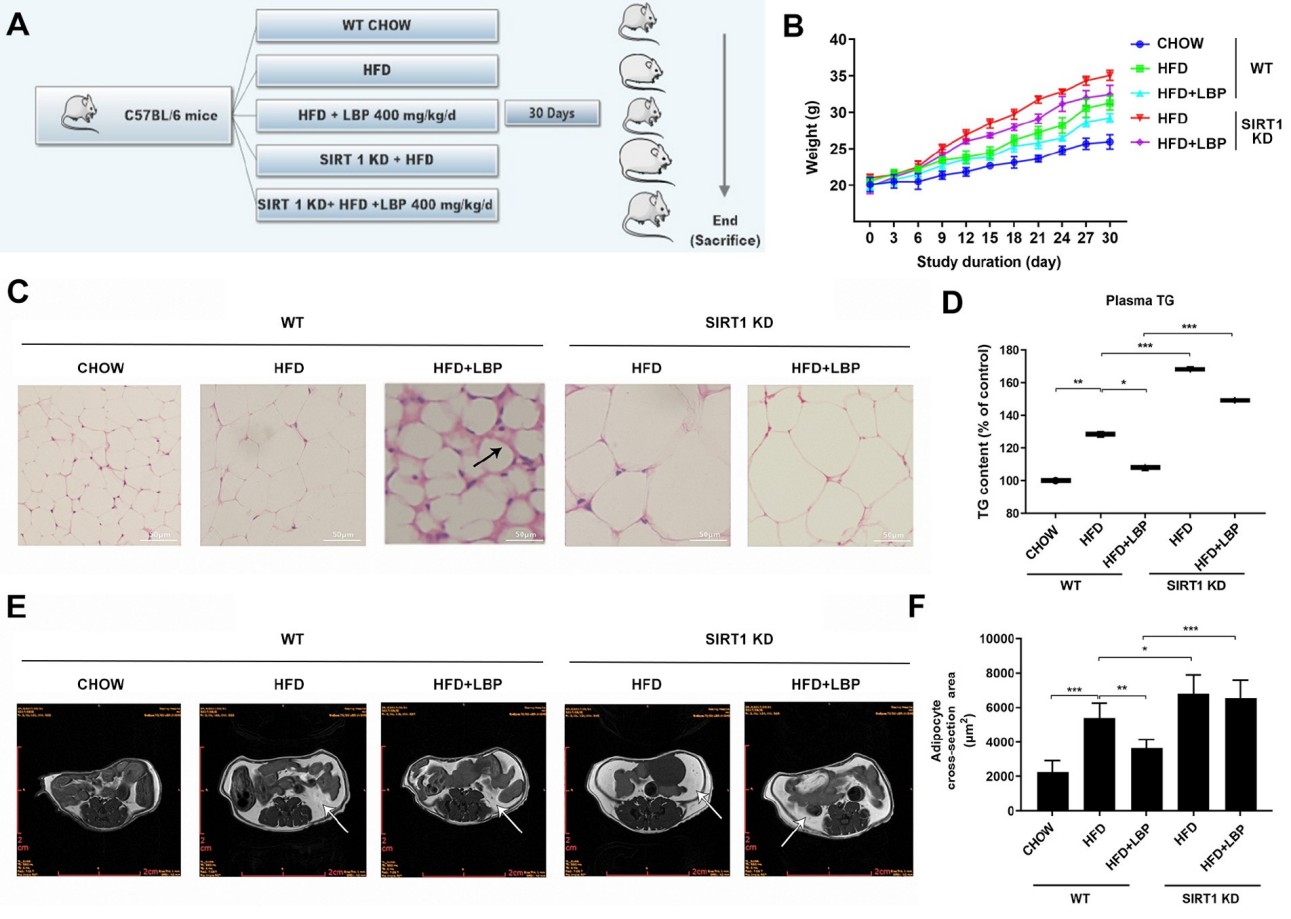
Figure 2 LBP reduces lipid accumulation in adipose tissue through SIRT1 in a short-term HFD-fed mouse model (A) Initially, 4- to 6-week-old male C57BL/6 mice ( n=5) were fed with a CHOW diet or a HFD for 2 weeks. After 30 days, HFD-fed mice were gavaged with or without LBP (400 mg/kg/day) or with or without a Tail vein injection of the pAAV-ZsGreen-shRNA-mSIRT1 plasmid once every five days (arrowheads). (B) Mouse body weight. (C) H&E staining of mouse adipose tissue specimens. Scale bar: 50 μm. (D) Plasma TG level was detected with a TG content detection kit. (E) MR images of different groups. (F) The size of adipocytes, indicated by the cross-sectional surface area on randomly selected H&E-stained sections, was measured using ImageJ software. The quantitative data obtained after three repeated measurements are presented as the mean±SD. * P<0.05, ** P<0.01, and *** P<0.001.

For the first 2 weeks, mice were fed either CHOW or a HFD. Then, HFD-fed animals were gavaged for 30 days, with or without 400 mg/kg/day LBP. Moreover, the *shSIRT1* plasmid was either injected through the Tail vein every five days or not ( Figure 2A). The BW was measured every other day. Compared with control mice, HFD-fed mice exhibited increased BW, which was notably suppressed by LBP intervention ( Figure 2B). To clarify the role of SIRT1 in regulating adipose lipid storage as a result of LBP administration, *shSIRT1* was applied for the knockdown (KD) of *SIRT1* *in vivo*. As shown in Figure 2B, the BW of the HFD-fed wild-type (WT) mice that received LBP was lower than that of the corresponding group without LBP treatment. Moreover, compared with HFD-fed WT mice, HFD-fed SIRT1-KD mice gained more BW, whereas compared with HFD-fed WT mice, HFD-fed SIRT1-KD mice given LBP caused slight but not significant weight loss ( Figure 2B). Histological analysis revealed that supplementation of HFD-fed mice with 400 mg/kg/day LBP led to fewer and smaller adipocytes, as well as the occurrence of CLS, indicating a marked inhibitory effect of LBP on HFD-induced accumulation of adipocytes ( Figure 2C). Compared with CHOW diet feeding, HFD feeding led to a significant increase in plasma TG level ( Figure 2D). Furthermore, the LBP-induced decrease in TG level in HFD-fed WT mice was notably abolished in HFD-fed *SIRT1-*KD mice, suggesting that SIRT1 strongly impacts LBP-induced amelioration of lipid metabolism. MRI scans also suggested that the fat mass of HFD-fed *SIRT1*-KD mice was much greater than that of WT mice, whereas the LBP-induced increase in fat mass was abolished by *SIRT1* knockdown *in vivo* ( Figure 2E). Consistently, a larger size and increased number of adipocytes were observed in the AT of HFD-fed *SIRT1*-KD mice, whereas LBP-induced amelioration of LD accumulation in AT in HFD-fed mice was notably abrogated in *SIRT1*-KD mice ( Figure 2F). Overall, LBP administration can prevent HFD-induced lipid accumulation in AT, which is remarkably attenuated through *SIRT1* knockdown *in vivo*, suggesting the potential involvement of SIRT1 in LBP-induced amelioration of LD accumulation in AT.

### LBP represses 3T3-L1 adipocyte differentiation and reduces LD storage

**Figure 3:**
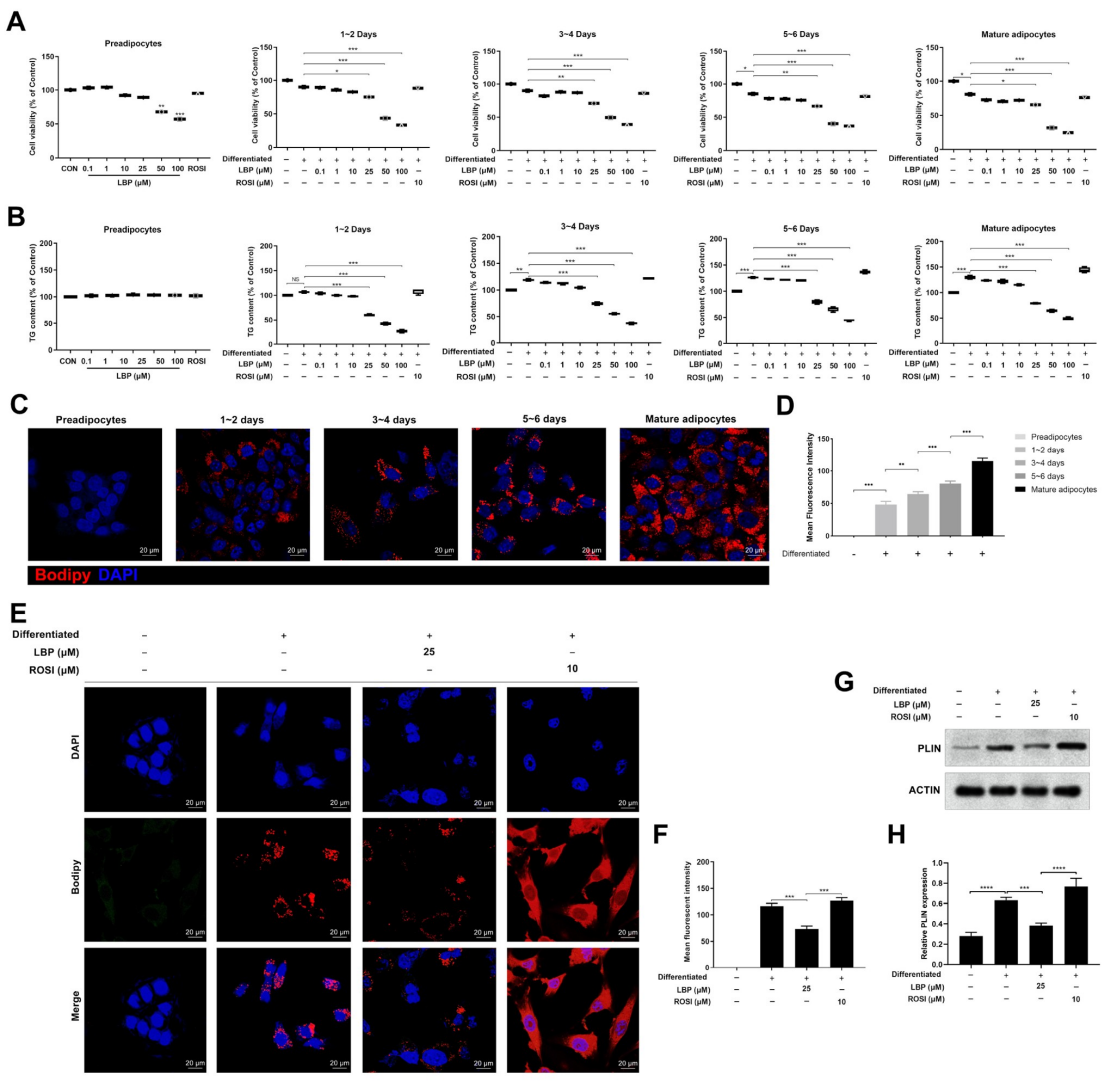
Figure 3 LBP represses 3T3-L1 adipocyte differentiation and reduces LD storage (A) Impacts of LBP on the activity of preadipocytes (PAs), as well as differentiated and mature adipocytes. 3T3-L1 PAs, differentiated or mature adipocytes, were administrated with LBP at various concentrations (0, 0.1, 1, 10, 25, 50, and 100 μM) for 24 h. 3T3-L1 cells were then induced to undergo differentiation with standard adipogenic medium supplemented with or without LBP dissolved in DMSO. CCK-8 colorimetric assay was used to determine cell viability after LBP intervention. The absorbance at 450 nm was measured, with the ordinate values representing optical density (OD) units. There were 6 replicates for each treatment. Rosiglitazone (ROSI) was used as a positive control. (B) Effect of LBP on lipid content in PAs, differentiated and mature adipocytes. The lipid content was measured using a triglyceride assay kit. The values were standardized to the total extractable protein. ROSI was used as a positive control. (C) After the labeling of LDs with BODIPY 558/568 (red), LD accumulation in adipocytes was visualized via microscopy using a 63× oil immersion objective. Scale bar: 20 μm. (D) The mean fluorescence intensity was measured. (E) LDs were labeled with BODIPY 558/568 (red), and LD accumulation in adipocytes was visualized after treatment with LBP and ROSI. Scale bar: 20 μm. (F) The mean fluorescence intensities were determined. (G,H) Western blot analysis of PLIN expression in adipocytes after treatment with LBP and ROSI. Data are shown as the mean±SD. * P<0.05, ** P<0.01, *** P<0.001, and **** P<0.0001. No statistical significance is denoted by NS.

The adipocyte cell model was established as previously described [ 30, 32] . At concentrations >50 μM, exposure to LBP for 24 h significantly inhibited the viability of PAs ( Figure 3A). During the differentiation period, cell viability was measured immediately after the differentiation medium was added each time. The results revealed that exposure to LBP decreased adipocyte viability in a dose-dependent manner. In the absence of LBP treatment, cell viability decreased on the 6 ^th^ day of differentiation, possibly due to increased terminal differentiation and rounding of adipocytes, which led to their floatation in the medium [35]. The viability of differentiating adipocytes and mature adipocytes remained unchanged after treatment with 25 μM LBP, revealing that LBP might have a stronger effect on the early stages of adipocyte differentiation. No marked change in TG content was detected in PAs after exposure to LBP for 24 h ( Figure 3B). Additionally, increased lipid accumulation was observed during differentiation. Moreover, considerably lower TG content was detected in the differentiating and mature cells treated with LBP at least 25 μM than in the cells without LBP treatment. In addition, as shown in Figure 3C,D, immunofluorescence experiments confirmed that the accumulation of LDs was greater in the differentiated group than in the control group. Thus, considering all the experiments performed, we confirmed that 25 μM LBP was appropriate for use to achieve the maximal biological effect in the subsequent experiments. In addition, Bodipy staining revealed less LD accumulation in LBP-treated cells than in differentiated medium-treated cells ( Figure 3E,F). Furthermore, PLIN (a lipid droplet-associated protein) expression was greater in the differentiated group than in the control group ( Figure 3G,H). LBP (25 μM) decreased the expression of PLIN in differentiated cells, and 10 μM ROSI was used as a positive control. Taken together, LBP intervention delayed differentiation and decreased LD accumulation in adipocytes.

### LBP inhibits LD-associated gene expression in mouse AT and adipocytes

**Figure 4:**
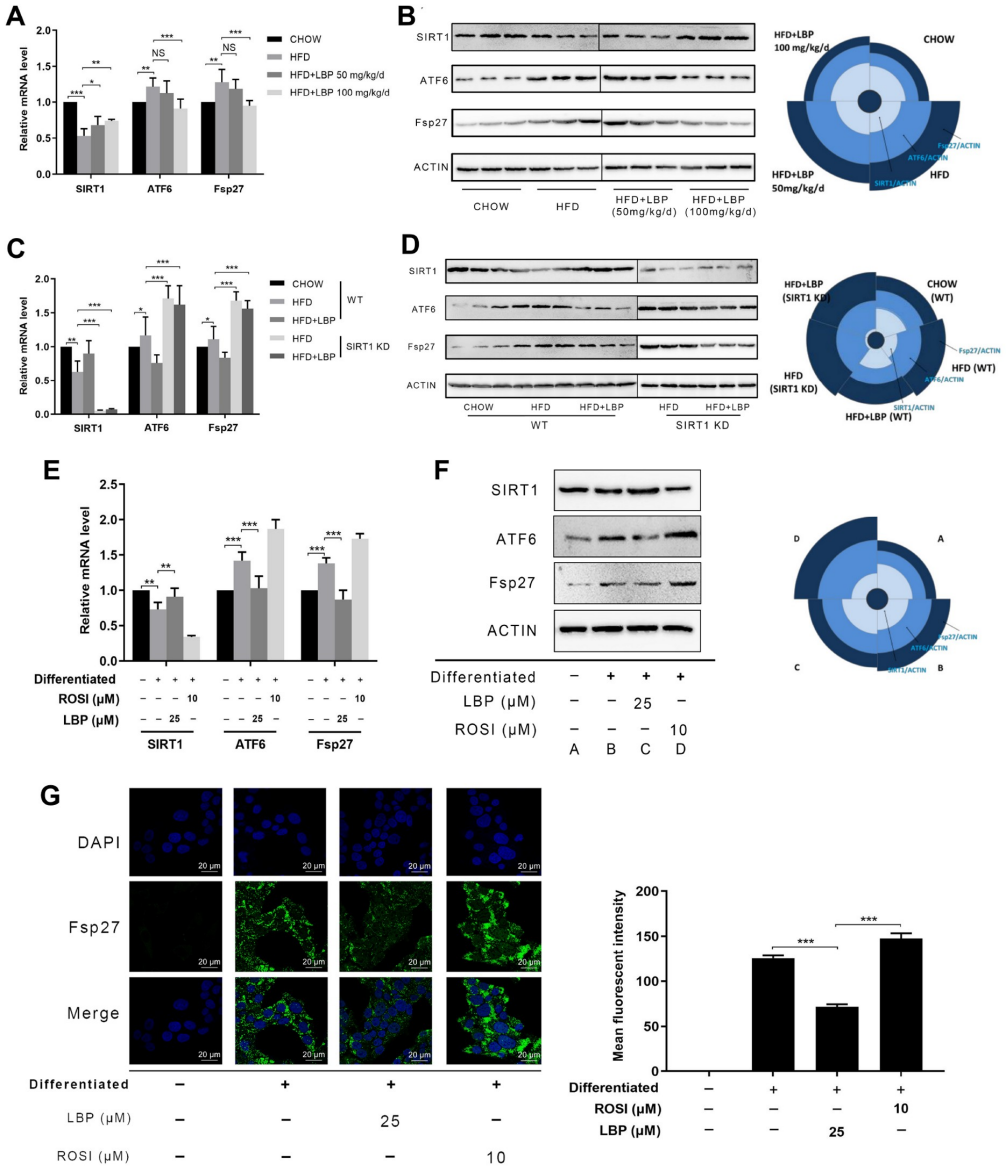
Figure 4 LBP inhibits the expressions of LD-associated genes in mouse adipose tissue and adipocytes (A) RT-qPCR analysis of SIRT1, ATF6 and Fsp27 mRNA expressions in adipocyte tissue from different groups. (B) Western blot analysis of SIRT1, ATF6 and Fsp27 protein levels relative to actin; Rose diagrams for quantification of the indicated genes. (C) RT-qPCR analysis of SIRT1, ATF6 and Fsp27 mRNA expressions in adipocyte tissue from WT and SIRT1-KD mice. (D) Western blot analysis of SIRT1, ATF6 and Fsp27 protein levels; Rose diagrams for quantification of the indicated genes. (E) The mRNA levels of the indicated genes in adipocytes were measured by RT-qPCR. (F) Western blot analysis of the protein expressions of the indicated genes relative to actin in adipocytes. Rose diagrams for quantification of the indicated genes. (G) Visualization of Fsp27 expression in adipocytes via confocal microscopy using a 63× oil immersion objective. Scale bar: 20 μm. The mean fluorescence intensity was determined. Data are shown as the mean±SD. * P<0.05, ** P<0.01, and *** P<0.001. No statistical significance is denoted by NS.

LBP administration ameliorated LD accumulation in mice fed with a HFD and decreased lipid accumulation in adipocytes. Next, we explored the effect of LBP on LD-associated genes in mouse AT and adipocytes. We found that HFD reduced the mRNA and protein levels of SIRT1 in AT, which were significantly inhibited by LBP administration ( Figure 4A,B). The LD-associated genes *ATF6* and *Fsp27* in HFD-fed mouse AT were notably increased at the mRNA and protein levels but were significantly inhibited by LBP administration. However, the LBP-induced decrease in ATF6 and Fsp27 was abrogated by *SIRT1* knockdown in mice, suggesting that the inhibition of LD-associated genes by SIRT1 is important and is followed by LBP administration ( Figure 4C,D). Furthermore, we investigated the effects of LBP on LD-associated genes in 3T3-L1 adipocytes. At both the mRNA and protein levels, a significant increase in SIRT1 was observed in 3T3-L1 adipocytes treated with LBP ( Figure 4E,F), accompanied by enhanced SIRT1 activity. However, LBP treatment reduced Fsp27 mRNA and protein levels in 3T3-L1 adipocytes compared with those in control adipocytes. Immunofluorescence cytochemistry further confirmed decreased intracellular Fsp27 expression in 3T3-L1 adipocytes following LBP intervention ( Figure 4G), suggesting that SIRT1 plays an important role in LBP-induced suppression of Fsp27 expression. Additionally, the mRNA and protein expressions of ATF6 were notably upregulated in mature adipocytes but were dramatically inhibited by LBP treatment. Collectively, these results suggest that LBP exerts an inhibitory effect on LD-associated Fsp27 and ATF6 in ATs and adipocytes, which might be associated with SIRT1.

### LBP inhibits LBP-induced LD accumulation in adipocytes

**Figure 5:**
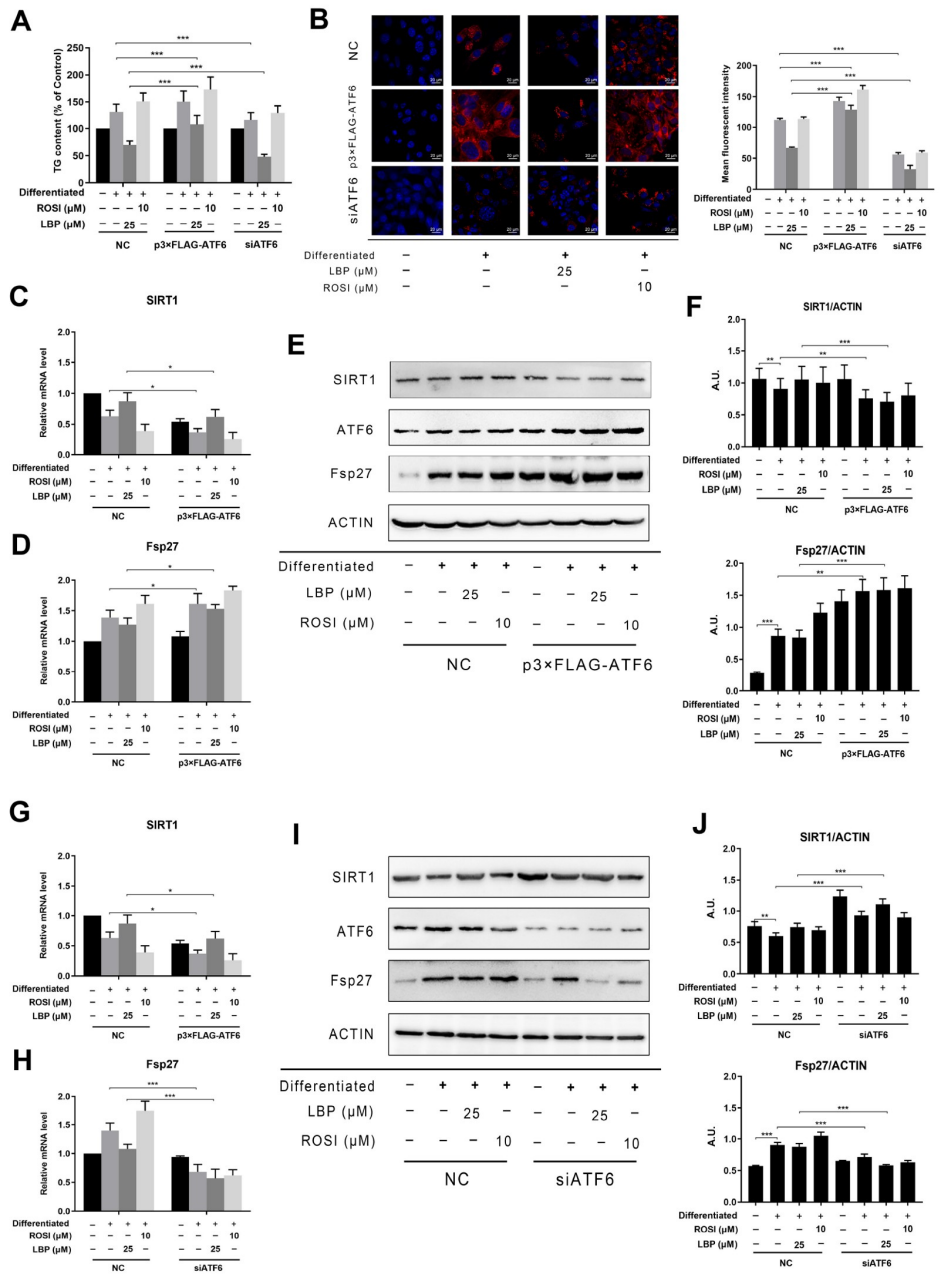
Figure 5 ATF6 participates in the LBP-induced decrease in LD accumulation in adipocytes (A) After transfection with the p3×FLAG-ATF6 plasmid, siRNA targeting ATF6, or negative control vector, mature adipocytes were treated with LBP (25 μM). A TG content detection kit was used to detect the intracellular TG content. (B) LD accumulation in adipocytes was visualized microscopically using a 63× oil immersion objective after transfection with the p3×FLAG-ATF6 plasmid, siRNA targeting ATF6, or negative control vector. Scale bar: 20 μm. The mean fluorescence intensity was determined. (C,D) The relative mRNA levels of the indicated genes were tested by RT-qPCR after transfection with the p3×FLAG-ATF6 plasmid or its corresponding control vector. (E) The protein levels of the indicated genes relative to those of actin were examined by western blot analysis after transfection with the p3×FLAG-ATF6 plasmid or its corresponding control vector. (F) Bar charts for quantification of the indicated proteins. (G,H) The relative mRNA levels of genes were measured by RT-qPCR after transfection of cells with siRNA targeting ATF6 or its corresponding control siRNA. (I) The protein levels of genes relative to that of actin were detected by western blot analysis after transfection with siRNA targeting ATF6 or the corresponding control siRNA. (J) Bar charts for quantification of the indicated proteins. Data are shown as the mean±SD.* P<0.05, ** P<0.01, and *** P<0.001.

ATF6 is a novel cellular signal metabolism regulator that can modulate lipid storage and obesity. Our findings indicated that the effect of LBP on SIRT1 mRNA and protein in both ATs and adipocytes was opposite to that of ATF6. Thus, we hypothesized that ATF6 might be closely associated with SIRT1 in LBP-induced LD accumulation in adipocytes. However, the underlying mechanism is still unknown. Then, 3T3-L1 adipocytes were transfected with the p3×FLAG-ATF6 overexpression plasmid, and whether ATF6 is involved in LBP-induced amelioration of LD accumulation in adipocytes was determined. As expected, compared with the control vector, ATF6 overexpression suppressed the LBP-induced decrease in TG content in 3T3-L1 adipocytes ( Figure 5A). Moreover, the LBP-induced decrease in TG content in 3T3-L1 adipocytes was enhanced by ATF6 siRNA transfection in 3T3-L1 adipocytes compared with that in 3T3-L1 adipocytes transfected with the control siRNA ( Figure 5A). Next, as measured using Bodipy staining, ATF6 overexpression plasmid transfection led to greater LD accumulation in LBP-induced 3T3-L1 adipocytes than transfection with the control vector ( Figure 5B). However, compared with control siRNA transfection, ATF6 siRNA transfection resulted in a greater decrease in LD accumulation in LBP-treated 3T3-L1 adipocytes ( Figure 5C). These results suggested the involvement of ATF6 in LBP-induced suppression of LD accumulation in adipocytes. According to the RT-qPCR and western blot analysis results, ectopic ATF6 expression notably reduced SIRT1 level and elevated Fsp27 level in 3T3-L1 adipocytes following LBP treatment ( Figure 5C–F). However, SIRT1 expression at the mRNA and protein levels was elevated in LBP-induced adipocytes after silencing of *ATF6*, suggesting that ATF6 may act as a negative modulator of SIRT1 ( Figure 5G–J). Additionally, silencing of *ATF6* was accompanied by downregulation of the LD-associated gene Fsp27 expression in LBP-induced 3T3-L1 adipocytes. Overall, ATF6 is essential for the LBP-induced suppression of LD accumulation, as well as the levels of SIRT1 and certain LD-associated genes, such as Fsp27, in adipocytes.

### LBP stimulates ATF6-dependent SIRT1 transcription in adipocytes

**Figure 6:**
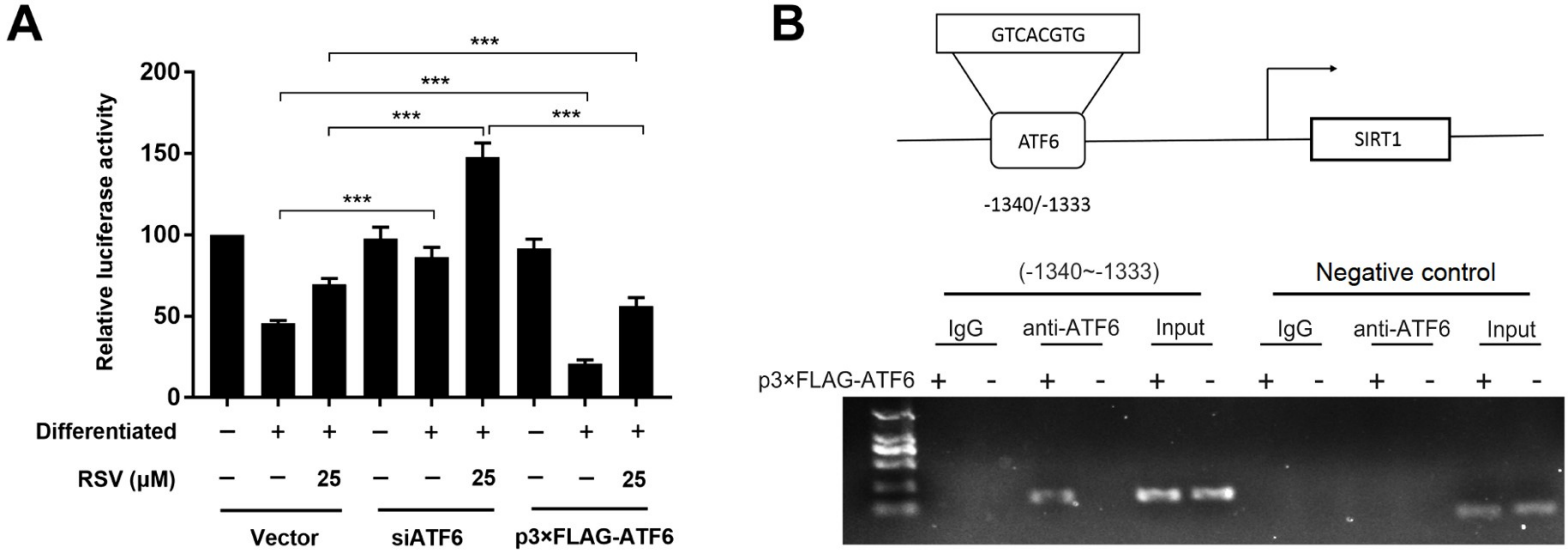
Figure 6 LBP induces ATF6-dependent *SIRT1* transcription in adipocytes (A) Adipocytes were subjected to the indicated treatments, and SIRT1 promoter activity was determined by dual-luciferase reporter assay after siRNA for ATF6 or p3×FLAG-ATF6 plasmid transfection. (B) Schematic diagram of the human SIRT1 gene promoter showing the core promoter sequence used for ChIP. The direct binding of ATF6 to the SIRT1 promoter was analyzed by ChIP. Chromatin DNA fragments were immunoprecipitated with an anti-ATF6 antibody using IgG as a negative control, followed by isolation and RT-qPCR amplification of the precipitated DNA, with the input (total DNA extracted) as a positive control and GAPDH as a internal control. Data are shown as the mean±SD. *** P<0.001.

Next, we explored the potential regulatory mechanism underlying SIRT1 and ATF6 in adipocytes in the context of LBP intervention. To determine the ability of ATF6 to regulate SIRT1 transcription, adipocytes were transfected with the *pGL3-Basic-SIRT1* plasmid with firefly luciferase activity, followed by cotransfection with siRNA against *ATF6* or the p3×FLAG-ATF6 plasmid and pRL-TK plasmid. According to the results of the dual luciferase assay, silencing of *ATF6* markedly enhanced LBP-induced SIRT1 transcriptional activity, with opposite results observed when ATF6 was overexpressed ( Figure 6A). These results indicated that the regulation of SIRT1 mRNA transcription by LBP treatment might be mediated by ATF6. To further determine whether ATF6 represses SIRT1 mRNA expression by directly binding to its promoter, we verified the site at which ATF6 binds to the SIRT1 promoter. In addition, ChIP assay was performed in adipocytes transfected with the p3×FLAG-ATF6 plasmid. The results showed that anti-ATF6 antibody precipitated with the promoter DNA of *SIRT1*, suggesting that endogenous ATF6 and SIRT1 could co-localize in the proximal region of the *SIRT1* promoter ( Figure 6B). Thus, LBP stimulates *SIRT1* transcription through direct binding of ATF6 to the *SIRT1* promoter in adipocytes.

## Discussion

**Figure 7:**
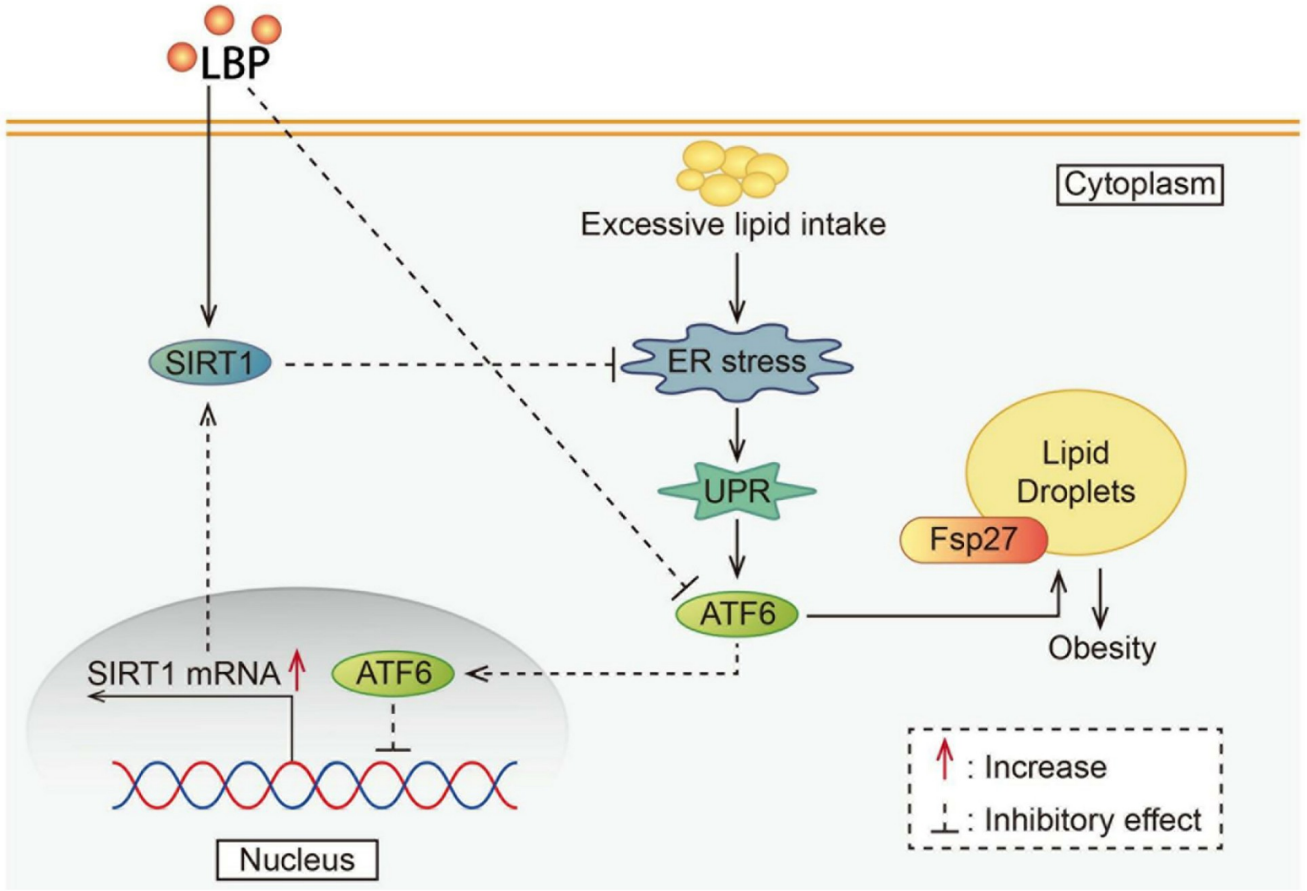
Figure 7 A schematic diagram showing that LBP attenuates LD accumulation via an ATF-6-dependent pathway

There is growing evidence that LD accumulation is a leading cause of obesity and is harmful to metabolic health [36]. Recent research highlights the link between the uncontrolled accumulation of LDs and the development of multiple diseases ( *e.g*., obesity, diabetes mellitus, neoplastic diseases, fatty liver diseases, and cardiovascular and neurodegenerative disorders) [ 11, 37– 42] . However, the understanding of the mechanism that promotes the metabolism of LDs is limited. This study provides the first *in vivo* and *in vitro* evidence that LBP alleviates LD accumulation by regulating Fsp27 via an ATF6/SIRT1-dependent mechanism ( Figure 7). LBP administration led to a decrease in the BW of mice, which was associated with reduced LD accumulation in AT. LBP-induced fat loss was closely related to the interaction between SIRT1 and ATF6. Furthermore, LBP inhibited ATF6 expression and relieved the ATF6-mediated inhibition of SIRT1 mRNA transcription, contributing to the enhanced inhibition of ER stress and ultimately suppressing ATF6 activation.

Our present study demonstrated that BW and TG levels in mice were positively associated with the administered dose of LBP. The beneficial effect was more significant in mice administered with 100 mg/kg/day LBP than in those administered with 50 mg/kg/day LBP. A previous study revealed that mice receiving a supplemented diet (HFD, with or without 20 or 40 mg of LBP) for 5 weeks exhibited a significant reduction in BW [6]. Consistent with these results, we provided evidence that *SIRT1*-KD mice fed with a HFD and treated with LBP (400 mg/kg/day) for 30 days exhibited slight decreases in BW and TG content. However, a much greater dose of LBP did not result in a significant reduction in the WT group as expected, suggesting that the duration of treatment may affect the effect of LBP. In the first experiment, mice were treated with a HFD and LBP at the same time, whereas in the second experiment, the mice were fed with a HFD for 2 weeks, indicating that LBP intake might be necessary as early as possible. The most important aim of this animal model was to confirm the critical role of SIRT1 in LBP-induced BW reduction. During the 30-day period, the benefits of LBP were nearly abolished without SIRT1. Hence, the discrepancies found in the present study can be attributed to differences in various aspects of the experimental design, namely, the length of the experimental period, the dose of LBP, the mode of LBP administration, the animal strain and, most importantly, the status of SIRT1.

We demonstrated that LBP markedly decreased TG level in HFD-fed mice. TGs are known to be synthesized in the ER and fill the core of growing LDs [43]. LDs are lipid storage depots, and their dynamic alterations, such as biogenesis and growth, are of critical importance in modulating lipid homeostasis. Lipolysis facilitates mitochondrial biogenesis and oxidative metabolism via a SIRT1-dependent pathway [44]. Here, we showed that Fsp27, a fat-specific protein 27 located at LD-LD contact sites, is essential for controlling LD growth and fusion, resulting in LD accumulation and obesity [ 10, 45] . Similarly, evidence suggests that AT is a major target mediating the metabolic effect of Fsp27. We observed a decrease in Fsp27 expression in the AT of HFD-fed mice treated with LBP. The ER regulates diverse cellular processes involving inflammation, insulin signaling and nutrient metabolism via the UPR [46]. Persistent activation of the UPR has been implicated in the nosogenesis of multiple metabolic disorders [ 47, 48] . Chronic UPR activation has been observed in the liver and/or AT of dietary and genetically obese murine models, as well as in obese populations and those with nonalcoholic fatty liver disease. ATF6 was upregulated in the presence of HFD, indicating that HFD contributes to the activation of the UPR. In this study, LBP significantly downregulated the activity of ATF6 in HFD-fed mice. Conversely, the LD-accumulating effects of Fsp27 were enhanced, and the fat-lowering effect of LBP was abrogated in *SIRT1*-KD mice. Therefore, we assume that SIRT1 may act as a critical regulator of LBP-induced LD reduction, which might further ameliorate the UPR and ER stress.

In accordance with previous observations, adipocytes incubated with 400 μg/mL LBP in culture medium were found to exhibit inhibited adipogenesis [49]. In both 3T3-L1 maturing and mature adipocytes, LBP significantly decreased cell viability and decreased lipid accumulation and intracellular TG level in a dose-dependent manner. Regarding 3T3-L1 differentiating PAs, exposure to 0.1 and 10 μM LBP did not modify the TG content. In contrast, 25 μM LBP led to a significant reduction. Moreover, earlier exposure of 3T3-L1 adipocytes to LBP triggered a much more significant reduction in the intracellular TG content. This result was consistent with the *in vivo* results.

The expressions of SIRT1, ATF6 and Fsp27, which are LD-related proteins, were analyzed in mature adipocytes treated with 25 μM LBP. *In vivo* and *in vitro*, it significantly reduced ATF6 and Fsp27 at the mRNA and protein levels but enhanced SIRT1 activity as expected. ATF6 is essential for the LBP-mediated reduction in LD accumulation. First, ATF6 is a UPR signaling factor that promotes LD growth, as its depletion dramatically decreases LD accumulation in mature adipocytes, and its ectopic expression evidently promotes LD accumulation. LBP has been proposed to drive its beneficial effects by targeting and activating SIRT1, while several researchers have surprisingly demonstrated that the conventional ER stress-activated transcription factor ATF6 seems to be dispensable for the induction of SIRT1 [50]. Here, we found that ATF6 is indispensable for inhibiting the expression of SIRT1. ATF6 silencing promoted the upregulation of SIRT1 level, whereas ATF6 overexpression led to SIRT1 deficiency.

Based on the above evidence, it is hypothesized that ATF6 may have a transcriptional effect on SIRT1. ATF6, which is a bZIP transcription factor of the ATF subfamily [51], is expected to be either a transcriptional activator or a repressor. ATF6 is suggested to confer a multidrug resistance phenotype to gastric cancer cells by deactivating SIRT1 [52]. We identified a potential binding site for ATF6 in the *SIRT1* promoter region through direct binding to which ATF6 might directly deactivate SIRT1 transcriptional activity.

In summary, our work provides strong evidence that LBP supplements are beneficial for reducing LD accumulation in both ATs and adipocytes, depending on the concentration, duration and method of LBP treatment. Furthermore, SIRT1 was identified as a critical factor that modulates the LBP-induced fat-lowering process. Our work provides novel insight into the mechanism by which ATF6, as a transcriptional repressor, alleviates SIRT1 activity. Our study raises the possibility that LBP exerts its effects not only by targeting SIRT1 but also by targeting ATF6, which will further ameliorate the UPR and ER stress and may represent a therapeutic strategy for obesity.
